# Bis[(2-amino­phen­yl)methanol-κ^2^ *N*,*O*]bis­(nitrato-κ*O*)cobalt(II)

**DOI:** 10.1107/S1600536810032381

**Published:** 2010-08-18

**Authors:** Majid Esmhosseini, Mahdokt Rezazadeh

**Affiliations:** a Department of Chemistry, University of Urmiyeh, Urmyieh, Iran

## Abstract

The asymmetric unit of the title compound, [Co(NO_3_)_2_(C_7_H_9_NO)_2_], contains one-half of the mol­ecule. The Co^II^ atom (site symmetry 2) is six-coordinate in a distorted octahedral configuration bonded by two N and two O atoms from two (2-amino­phen­yl)methanol ligands and two O atoms from the two nitrate anions. Crystal packing is stabilized by inter­molecular N—H⋯O, O—H⋯O and C—H⋯O hydrogen-bonding inter­actions.

## Related literature

For related structures with different metal atoms, see: Bandoli *et al.* (2002 ▶); Lewiriski *et al.* (1998 ▶); Esmhosseini (2010 ▶); Esmhosseini & Maleki (2010 ▶). For bond distances and angles, see: Allen (2002 ▶).
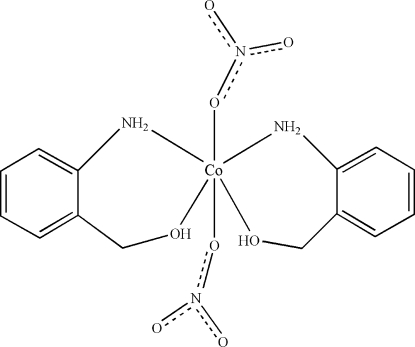


## Experimental

### 

#### Crystal data

[Co(NO_3_)_2_(C_7_H_9_NO)_2_]
*M* *_r_* = 429.25
Orthorhombic, 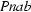

*a* = 7.2554 (6) Å
*b* = 10.1685 (7) Å
*c* = 23.250 (2) Å
*V* = 1715.3 (3) Å^3^
*Z* = 4
Mo *K*α radiation
μ = 1.05 mm^−1^
*T* = 120 K
0.50 × 0.20 × 0.15 mm

#### Data collection

Bruker SMART CCD area-detector diffractometer
Absorption correction: multi-scan (*SADABS*; Bruker, 2003 ▶) *T*_min_ = 0.768, *T*_max_ = 0.861
6514 measured reflections
2271 independent reflections
2065 reflections with *I* > 2σ(*I*)
*R*_int_ = 0.046

#### Refinement

*R*[*F* ^2^ > 2σ(*F* ^2^)] = 0.032
*wR*(*F* ^2^) = 0.088
*S* = 1.08
2271 reflections
135 parameters
H atoms treated by a mixture of independent and constrained refinement
Δρ_max_ = 0.46 e Å^−3^
Δρ_min_ = −0.46 e Å^−3^

### 

Data collection: *SMART* (Bruker, 1998 ▶); cell refinement: *SAINT* (Bruker, 1998 ▶); data reduction: *SAINT*; program(s) used to solve structure: *SHELXTL* (Sheldrick, 2008 ▶); program(s) used to refine structure: *SHELXTL*; molecular graphics: *ORTEP-3 for Windows* (Farrugia, 1997 ▶); software used to prepare material for publication: *WinGX* (Farrugia, 1999 ▶).

## Supplementary Material

Crystal structure: contains datablocks global, I. DOI: 10.1107/S1600536810032381/jj2052sup1.cif

Structure factors: contains datablocks I. DOI: 10.1107/S1600536810032381/jj2052Isup2.hkl

Additional supplementary materials:  crystallographic information; 3D view; checkCIF report

## Figures and Tables

**Table:** 

O2—Co1	2.1288 (11)
Co1—O1^i^	2.1025 (10)
Co1—N1	2.1463 (12)

**Table:** 

O1—Co1—O1^i^	84.64 (6)
O1—Co1—O2	88.90 (4)
O1^i^—Co1—O2	84.43 (4)
O2—Co1—O2^i^	170.98 (5)
O1—Co1—N1^i^	169.09 (5)
O1^i^—Co1—N1^i^	85.27 (4)
O2—Co1—N1^i^	94.26 (4)
O1—Co1—N1	85.27 (4)
O2—Co1—N1	91.22 (5)
N1^i^—Co1—N1	105.07 (7)

Symmetry code: (i) 
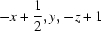
.

**Table 2:** Hydrogen-bond geometry (Å, °)

*D*—H⋯*A*	*D*—H	H⋯*A*	*D*⋯*A*	*D*—H⋯*A*
N1—H1*C*⋯O3^ii^	0.88 (2)	2.15 (2)	2.9897 (16)	158 (2)
N1—H1*D*⋯O4^iii^	0.84 (3)	2.24 (3)	3.0689 (17)	169 (2)
O1—H1*E*⋯O2^iv^	0.84 (2)	1.86 (2)	2.6908 (14)	172 (2)
C1—H1*B*⋯O4^iii^	0.97	2.54	3.4145 (18)	151

Symmetry codes: (ii) 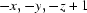; (iii) 
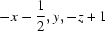
; (iv) 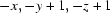.

**Fig. 1.**
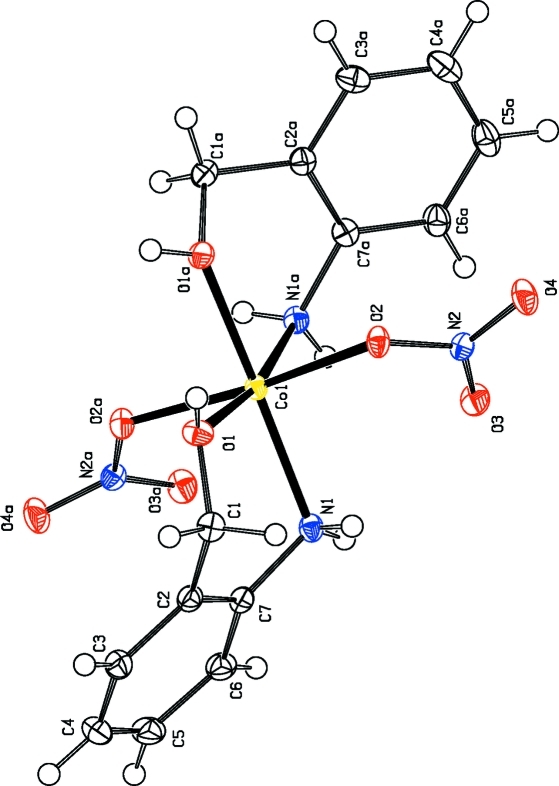
The molecular structure of the title molecule, [Co(C7H9NO)2(NO3)2], with the atom-numbering scheme. Displacement ellipsoids are drawn at the 50% probability level.

**Fig. 2.**
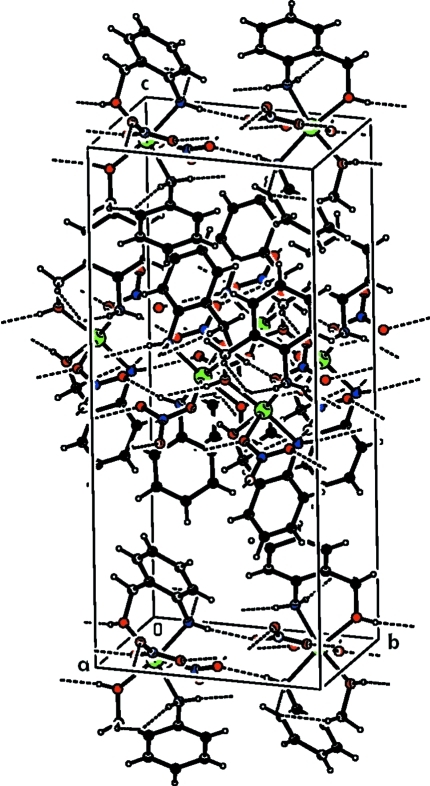
Unit-cell packing diagram for title molecule, [Co(C7H9NO)2(NO3)2]. Hydrogen bonds are shown as dashed lines.

## supplementary crystallographic information

### Comment

Only four metal complexes with the (2-aminophenyl)methanol, bidentate ligand have been prepared and include the Re (Bandoli *et al.*, 2002), Al (Lewiriski *et al.*, 1998), Zn (Esmhosseini, 2010) and Mn (Esmhosseini & Maleki, 2010) compounds. We report herein the synthesis and crystal structure of the Co analogue compound, [Co(C_7_H_9_NO)_2_(NO_3_)_2_].

The asymmetric unit of the title compound, [Co(C_7_H_9_NO)_2_(NO_3_)_2_], contains one-half of the molecule (Fig. 1). The Co^II^ atom in the cation is six-coordinate in a distorted hexagonal configuration bonded by two N and two O atoms from two (2-aminophenyl)methanol ligands and two O atoms from the two nitrate anions.  Bond distances and angles are in normal ranges (Allen, 2002).  Intermolecular N—H···O, O—H···O and C—H···O hydrogen bonding stabilize the crystal structure, (Table 2, Fig. 2).

### Experimental

For the preparation of the title compound, a solution of (2-aminophenyl)methanol (0.25 g, 2.00 mmol) in methanol (10 ml) was added to a solution of Co(NO_3_)_2_.6H_2_O (0.29 g, 1.00 mmol) in methanol (10 ml) and the resulting colorless solution was stirred for 20 min at 313 K.  This solution was left to evaporate slowly at room temperature.  After one week, light violet block crystals of the title compound were isolated (yield 0.32 g, 74.5%).

### Refinement

The H atoms on the C and N atoms were positioned geometrically, and refined as riding atoms, with C–H = 0.93 Å (CH), C–H = 0.97 Å (CH_2_), N–H = 0.88, 0.84 Å (NH_2_) and constrained to ride on their parent atoms, with *U*_iso_(H) = 1.2*U*_eq_(C), *U*_iso_(H) = 1.9*U*_eq_(N). The H on O1 was located by a Fourier map and refined isotropically.

### Crystal data

**Table:**

[Co(NO_3_)_2_(C_7_H_9_NO)_2_]	*F*(000) = 884
*M**_r_* = 429.25	*D*_x_ = 1.662 Mg m^−^^3^
Orthorhombic, *P**n**a**b*	Mo *K*α radiation, λ = 0.71073 Å
Hall symbol: -P 2bc 2n	Cell parameters from 1009 reflections
*a* = 7.2554 (6) Å	θ = 2.2–29.2°
*b* = 10.1685 (7) Å	µ = 1.05 mm^−^^1^
*c* = 23.250 (2) Å	*T* = 120 K
*V* = 1715.3 (3) Å^3^	Block, light violet
*Z* = 4	0.50 × 0.20 × 0.15 mm

### Data collection

**Table:**

Bruker SMART CCD area-detector diffractometer	2271 independent reflections
Radiation source: fine-focus sealed tube	2065 reflections with *I* > 2σ(*I*)
graphite	*R*_int_ = 0.046
φ and ω scans	θ_max_ = 29.2°, θ_min_ = 2.2°
Absorption correction: multi-scan (*SADABS*; Bruker, 2003)	*h* = −6→9
*T*_min_ = 0.768, *T*_max_ = 0.861	*k* = −11→13
6514 measured reflections	*l* = −26→31

### Refinement

**Table:**

Refinement on *F*^2^	Primary atom site location: structure-invariant direct methods
Least-squares matrix: full	Secondary atom site location: difference Fourier map
*R*[*F*^2^ > 2σ(*F*^2^)] = 0.032	Hydrogen site location: inferred from neighbouring sites
*wR*(*F*^2^) = 0.088	H atoms treated by a mixture of independent and constrained refinement
*S* = 1.08	*w* = 1/[σ^2^(*F*_o_^2^) + (0.0456*P*)^2^ + 0.8425*P*] where *P* = (*F*_o_^2^ + 2*F*_c_^2^)/3
2271 reflections	(Δ/σ)_max_ = 0.007
135 parameters	Δρ_max_ = 0.46 e Å^−^^3^
0 restraints	Δρ_min_ = −0.46 e Å^−^^3^

### Special details

**Table:**

Geometry. All e.s.d.'s (except the e.s.d. in the dihedral angle between two l.s. planes) are estimated using the full covariance matrix. The cell e.s.d.'s are taken into account individually in the estimation of e.s.d.'s in distances, angles and torsion angles; correlations between e.s.d.'s in cell parameters are only used when they are defined by crystal symmetry. An approximate (isotropic) treatment of cell e.s.d.'s is used for estimating e.s.d.'s involving l.s. planes.
Refinement. Refinement of *F*^2^ against ALL reflections. The weighted *R*-factor *wR* and goodness of fit *S* are based on *F*^2^, conventional *R*-factors *R* are based on *F*, with *F* set to zero for negative *F*^2^. The threshold expression of *F*^2^ > σ(*F*^2^) is used only for calculating *R*-factors(gt) *etc*. and is not relevant to the choice of reflections for refinement. *R*-factors based on *F*^2^ are statistically about twice as large as those based on *F*, and *R*- factors based on ALL data will be even larger.

### Fractional atomic coordinates and isotropic or equivalent isotropic displacement parameters (Å^2^)

**Table:**

	*x*	*y*	*z*	*U*_iso_*/*U*_eq_	
O3	−0.06393 (16)	0.10499 (10)	0.53584 (5)	0.0194 (2)	
O4	−0.24657 (15)	0.23434 (12)	0.58377 (5)	0.0213 (2)	
O1	0.18951 (16)	0.45350 (10)	0.44211 (4)	0.0155 (2)	
H1E	0.142 (3)	0.524 (2)	0.4531 (9)	0.029 (6)*	
C2	0.2247 (2)	0.32739 (15)	0.35482 (6)	0.0145 (3)	
C4	0.4195 (2)	0.26108 (18)	0.27496 (6)	0.0230 (3)	
H4	0.4794	0.2816	0.2407	0.028*	
N2	−0.11401 (17)	0.21530 (11)	0.55146 (5)	0.0135 (2)	
C7	0.23941 (19)	0.19961 (13)	0.37774 (7)	0.0143 (3)	
O2	−0.02236 (15)	0.31708 (9)	0.53328 (5)	0.0151 (2)	
Co1	0.2500	0.30062 (2)	0.5000	0.01128 (10)	
C6	0.3427 (2)	0.10409 (15)	0.34922 (6)	0.0179 (3)	
H6	0.3508	0.0195	0.3642	0.022*	
C3	0.3146 (2)	0.35577 (15)	0.30337 (6)	0.0188 (3)	
H3	0.3044	0.4396	0.2877	0.023*	
C5	0.4337 (2)	0.13548 (16)	0.29835 (7)	0.0223 (3)	
H5	0.5044	0.0721	0.2798	0.027*	
N1	0.15712 (18)	0.17223 (12)	0.43271 (5)	0.0140 (2)	
H1C	0.162 (3)	0.088 (2)	0.4413 (9)	0.028 (5)*	
H1D	0.044 (4)	0.189 (2)	0.4333 (10)	0.027 (6)*	
C1	0.1144 (2)	0.43142 (13)	0.38520 (6)	0.0158 (3)	
H1A	0.1181	0.5126	0.3633	0.019*	
H1B	−0.0131	0.4036	0.3882	0.019*	

### Atomic displacement parameters (Å^2^)

**Table:**

	*U*^11^	*U*^22^	*U*^33^	*U*^12^	*U*^13^	*U*^23^
O3	0.0226 (5)	0.0110 (4)	0.0246 (5)	−0.0008 (4)	0.0041 (4)	−0.0014 (4)
O4	0.0135 (5)	0.0244 (6)	0.0259 (6)	0.0003 (4)	0.0074 (4)	0.0004 (5)
O1	0.0207 (5)	0.0104 (4)	0.0152 (5)	0.0035 (4)	0.0008 (4)	−0.0007 (4)
C2	0.0126 (6)	0.0153 (6)	0.0155 (6)	−0.0010 (5)	−0.0010 (5)	−0.0010 (5)
C4	0.0208 (7)	0.0333 (8)	0.0149 (6)	−0.0045 (6)	0.0041 (6)	−0.0032 (6)
N2	0.0116 (5)	0.0129 (5)	0.0160 (5)	−0.0006 (4)	−0.0010 (4)	0.0005 (4)
C7	0.0129 (7)	0.0145 (6)	0.0155 (6)	−0.0022 (5)	−0.0001 (5)	−0.0014 (5)
O2	0.0134 (5)	0.0101 (4)	0.0219 (5)	−0.0008 (3)	0.0040 (4)	0.0008 (4)
Co1	0.01166 (16)	0.00913 (15)	0.01306 (15)	0.000	0.00220 (9)	0.000
C6	0.0174 (7)	0.0175 (6)	0.0190 (6)	0.0009 (5)	−0.0002 (5)	−0.0043 (5)
C3	0.0195 (7)	0.0214 (7)	0.0156 (6)	−0.0048 (6)	−0.0004 (6)	0.0016 (5)
C5	0.0195 (7)	0.0267 (8)	0.0206 (7)	0.0012 (6)	0.0027 (6)	−0.0092 (6)
N1	0.0141 (6)	0.0112 (5)	0.0168 (5)	−0.0004 (4)	0.0021 (4)	0.0007 (4)
C1	0.0151 (6)	0.0148 (6)	0.0175 (6)	0.0015 (5)	−0.0011 (5)	0.0016 (5)

### Geometric parameters (Å, °)

**Table:**

O3—N2	1.2338 (15)	O2—Co1	2.1288 (11)
O4—N2	1.2356 (16)	Co1—O1^i^	2.1025 (10)
O1—C1	1.4485 (17)	Co1—O2^i^	2.1288 (11)
O1—Co1	2.1025 (10)	Co1—N1^i^	2.1463 (12)
O1—H1E	0.83 (2)	Co1—N1	2.1463 (12)
C2—C3	1.393 (2)	C6—C5	1.392 (2)
C2—C7	1.408 (2)	C6—H6	0.9300
C2—C1	1.503 (2)	C3—H3	0.9300
C4—C5	1.392 (2)	C5—H5	0.9300
C4—C3	1.394 (2)	N1—H1C	0.88 (2)
C4—H4	0.9300	N1—H1D	0.84 (3)
N2—O2	1.3008 (15)	C1—H1A	0.9700
C7—C6	1.394 (2)	C1—H1B	0.9700
C7—N1	1.4378 (19)		
			
C1—O1—Co1	123.28 (8)	O1—Co1—N1	85.27 (4)
C1—O1—H1E	104.9 (15)	O1^i^—Co1—N1	169.09 (5)
Co1—O1—H1E	121.7 (14)	O2—Co1—N1	91.22 (5)
C3—C2—C7	118.74 (14)	O2^i^—Co1—N1	94.26 (4)
C3—C2—C1	120.48 (13)	N1^i^—Co1—N1	105.07 (7)
C7—C2—C1	120.78 (13)	C5—C6—C7	119.95 (14)
C5—C4—C3	119.28 (14)	C5—C6—H6	120.0
C5—C4—H4	120.4	C7—C6—H6	120.0
C3—C4—H4	120.4	C2—C3—C4	121.30 (15)
O3—N2—O4	123.42 (12)	C2—C3—H3	119.3
O3—N2—O2	118.50 (12)	C4—C3—H3	119.3
O4—N2—O2	118.08 (12)	C6—C5—C4	120.50 (14)
C6—C7—C2	120.22 (14)	C6—C5—H5	119.8
C6—C7—N1	120.72 (13)	C4—C5—H5	119.8
C2—C7—N1	118.92 (13)	C7—N1—Co1	113.56 (9)
N2—O2—Co1	122.01 (8)	C7—N1—H1C	112.0 (14)
O1—Co1—O1^i^	84.64 (6)	Co1—N1—H1C	114.4 (14)
O1—Co1—O2	88.90 (4)	C7—N1—H1D	112.5 (16)
O1^i^—Co1—O2	84.43 (4)	Co1—N1—H1D	100.0 (16)
O1—Co1—O2^i^	84.43 (4)	H1C—N1—H1D	103 (2)
O1^i^—Co1—O2^i^	88.90 (4)	O1—C1—C2	109.76 (11)
O2—Co1—O2^i^	170.98 (5)	O1—C1—H1A	109.7
O1—Co1—N1^i^	169.09 (5)	C2—C1—H1A	109.7
O1^i^—Co1—N1^i^	85.27 (4)	O1—C1—H1B	109.7
O2—Co1—N1^i^	94.26 (4)	C2—C1—H1B	109.7
O2^i^—Co1—N1^i^	91.22 (5)	H1A—C1—H1B	108.2
			
C3—C2—C7—C6	0.2 (2)	N1—C7—C6—C5	−174.82 (14)
C1—C2—C7—C6	−179.79 (13)	C7—C2—C3—C4	−0.8 (2)
C3—C2—C7—N1	175.93 (13)	C1—C2—C3—C4	179.16 (14)
C1—C2—C7—N1	−4.0 (2)	C5—C4—C3—C2	0.4 (2)
O3—N2—O2—Co1	−18.93 (17)	C7—C6—C5—C4	−1.3 (2)
O4—N2—O2—Co1	160.75 (10)	C3—C4—C5—C6	0.7 (2)
C1—O1—Co1—O1^i^	−174.89 (13)	C6—C7—N1—Co1	120.28 (12)
C1—O1—Co1—O2	−90.38 (11)	C2—C7—N1—Co1	−55.46 (15)
C1—O1—Co1—O2^i^	95.70 (11)	O1—Co1—N1—C7	49.56 (10)
C1—O1—Co1—N1^i^	162.6 (2)	O1^i^—Co1—N1—C7	72.1 (3)
C1—O1—Co1—N1	0.94 (11)	O2—Co1—N1—C7	138.36 (10)
N2—O2—Co1—O1	145.62 (10)	O2^i^—Co1—N1—C7	−34.48 (10)
N2—O2—Co1—O1^i^	−129.65 (10)	N1^i^—Co1—N1—C7	−126.90 (11)
N2—O2—Co1—N1^i^	−44.83 (10)	Co1—O1—C1—C2	−49.00 (15)
N2—O2—Co1—N1	60.38 (10)	C3—C2—C1—O1	−119.95 (14)
C2—C7—C6—C5	0.9 (2)	C7—C2—C1—O1	60.01 (17)

Symmetry codes: (i) −*x*+1/2, *y*, −*z*+1.

### Hydrogen-bond geometry (Å, °)

**Table:**

*D*—H···*A*	*D*—H	H···*A*	*D*···*A*	*D*—H···*A*
N1—H1C···O3^ii^	0.88 (2)	2.15 (2)	2.9897 (16)	158 (2)
N1—H1D···O4^iii^	0.84 (3)	2.24 (3)	3.0689 (17)	169 (2)
O1—H1E···O2^iv^	0.84 (2)	1.86 (2)	2.6908 (14)	172 (2)
C1—H1B···O4^iii^	0.97	2.54	3.4145 (18)	151.

Symmetry codes: (ii) −*x*, −*y*, −*z*+1; (iii) −*x*−1/2, *y*, −*z*+1; (iv) −*x*, −*y*+1, −*z*+1.
